# Caspase-mediated cleavage of the centrosomal proteins during apoptosis

**DOI:** 10.1038/s41419-018-0632-8

**Published:** 2018-05-11

**Authors:** Mi Young Seo, Kunsoo Rhee

**Affiliations:** Department of Biological Sciences, Seoul National University, Seoul, 08826 Korea

## Abstract

The centrosome is the major microtubule-organizing center and plays important roles in intracellular transport, cellular morphology, and motility. In mitotic cells, centrosomes function as spindle poles to pull a set of chromosomes into daughter cells. In quiescent cells, primary cilia are originated from the centrosomes. Given its involvement in various cellular processes, it is little surprising that the organelle would also participate in apoptotic events. However, it remains elusive how the centrosome changes in structure and organization during apoptosis. Apoptosis, a programmed cell death, is required for homeostatic tissue maintenance, embryonic development, stress responses, etc. Activation of caspases generates a cascade of apoptotic pathways, explaining much of what happens during apoptosis. Here, we report the proteolytic cleavage of selected centrosomal proteins in apoptotic cells. SAS-6, a cartwheel component of centrioles, was specifically cleaved at the border of the coiled-coil domain and the disordered C-terminus. Pericentrin, a scaffold of pericentriolar material, was also cleaved during apoptosis. These cleavages were efficiently blocked by the caspase inhibitors. We propose that the caspase-dependent proteolysis of the centrosomal proteins may destabilize the configuration of a centrosome. Loss of centrosomes may be required for the formation of apoptotic microtubule networks, which are essential for apoptotic fragmentation. This work demonstrates the first centrosomal targets by caspases during apoptosis.

## Introduction

The centrosome is the major microtubule-organizing center (MTOC) and consists of a pair of centrioles and the pericentriolar material (PCM). The centrioles assemble during S phase and segregate into daughter cells at the mitotic exit. SAS-6 is one of the core components important for centriole assembly and it is evolutionally conserved^1,2^. SAS-6 serves as a cartwheel protein of procentrioles^3–5^. The N-terminal domains of SAS-6 dimer self-assemble to make a ninefold symmetric ring and its coiled-coil domain constitutes the spoke radiating from the ring structure. The C-terminus of SAS-6 interacts with other proteins present in the centriolar walls. However, the detailed structure and function of the cartwheel among different species are not shared. Especially, mammalian SAS-6 cartwheel disassembles from the procentrioles during mitotic exit, while the centrioles in *Caenorhabditis elegans* and *Drosophila* retain the cartwheel component throughout the cell cycle^6,7^. Despite extensive research on its role in centriole formation, it remains to be elucidated how the release of SAS-6 from the centrioles is regulated in human cells and what would be the consequences if the cartwheel disassembly is triggered at any cell cyclic phase.

Pericentrin is one of the major PCM components and is important for the recruitment of other PCM proteins during early mitosis, ensuring the centrosome maturation and thus bipolar spindle formation^8–10^. The integrity of PCM is reported to be critical for maintaining centriole association during prolonged mitotic arrest^11,12^. Also, the separase-mediated cleavage of pericentrin is known to be the most critical event for centriole separation at the end of mitosis^13–15^. Therefore, the existence of the intact pericentrin determines not only the PCM integrity but also the centriolar configuration associated or separated, thus regulating the functional entity of the centrosome as a whole.

Apoptosis, a programmed cell death, is an important cellular event by which embryonic development, tissue organization, stress responses, immune reaction, and tumorigenesis are regulated at the multicellular level^16,17^. Apoptosis can also be intentionally triggered for chemical intervention of cancerous cells, making it a favorable targeted pathway for developing anticancer drugs^18–20^. The activation of caspases is the most important biochemical feature of apoptosis and initiates the demolition of cells at different phases^16,17^. Rather than all the cellular proteins being chopped simultaneously, there are certain pools of proteins that serve as the main targets for cleavages^21,22^. The target cellular structures of caspases include the cytoskeleton, the nucleus, ER, and Golgi. Cleavage of ROCK1 kinase by caspase-3 causes the membrane blebbing^23,24^. DNA fragmentation is a result of the activation of caspase-activated DNase (CAD)^25,26^. The disintegration of the nuclear envelope is a consequence of the proteolytic cleavages of nuclear lamins^27^. Caspase-dependent cleavages of GRASP65 are linked to Golgi fragmentation^28^. During the late phase of apoptosis, ER also fragments along with the cleavages of various translation initiation factors^21,29^. Rather than being a target of caspases, mitochondria release cytochrome C, which therefore activates the executioner caspases like caspase-3, 6, or 7^16,17,30^. Although the destructions of key cellular structures and organelles are reported as the morphological characteristics of apoptosis and mediated by targeting a certain pool of caspase substrates, it remains unclear how the centrosome changes in apoptotic cells, especially at the molecular details.

We hereby looked into whether there is any centrosomal change during apoptosis at the molecular level. Previous studies reported that the intensities of multiple centrosomal proteins reduced at specific phases of apoptosis: PCM components first and the centriolar components like centrin-2 later^31^. Cleavage of dynein, a known caspase target and a motor protein, might be one of the possible mechanisms explaining the reduced intensities of the centrosomal proteins by inhibiting the influx of the centrosomal proteins to the centrosome along the microtubules^31^. Alternatively, caspases may directly target centrosomal components for demolition of the centrosome. While centrosomal microtubules disassemble in the early phase of apoptosis, acentrosomal microtubules start to assemble during the execution phase of apoptosis. Such changes in microtubule network may be essential for the subsequent apoptotic events. Thus, the centrosome should be one of the target organelles for apoptotic proteases. This study demonstrates that SAS-6 and pericentrin are cleaved during apoptosis and the cleavages might be mediated by caspases, revealing the evidence of the centrosome as being the target of caspases during apoptosis.

## Results

### Cleavage of SAS-6 with the MG132 treatment

**Fig. 1:**
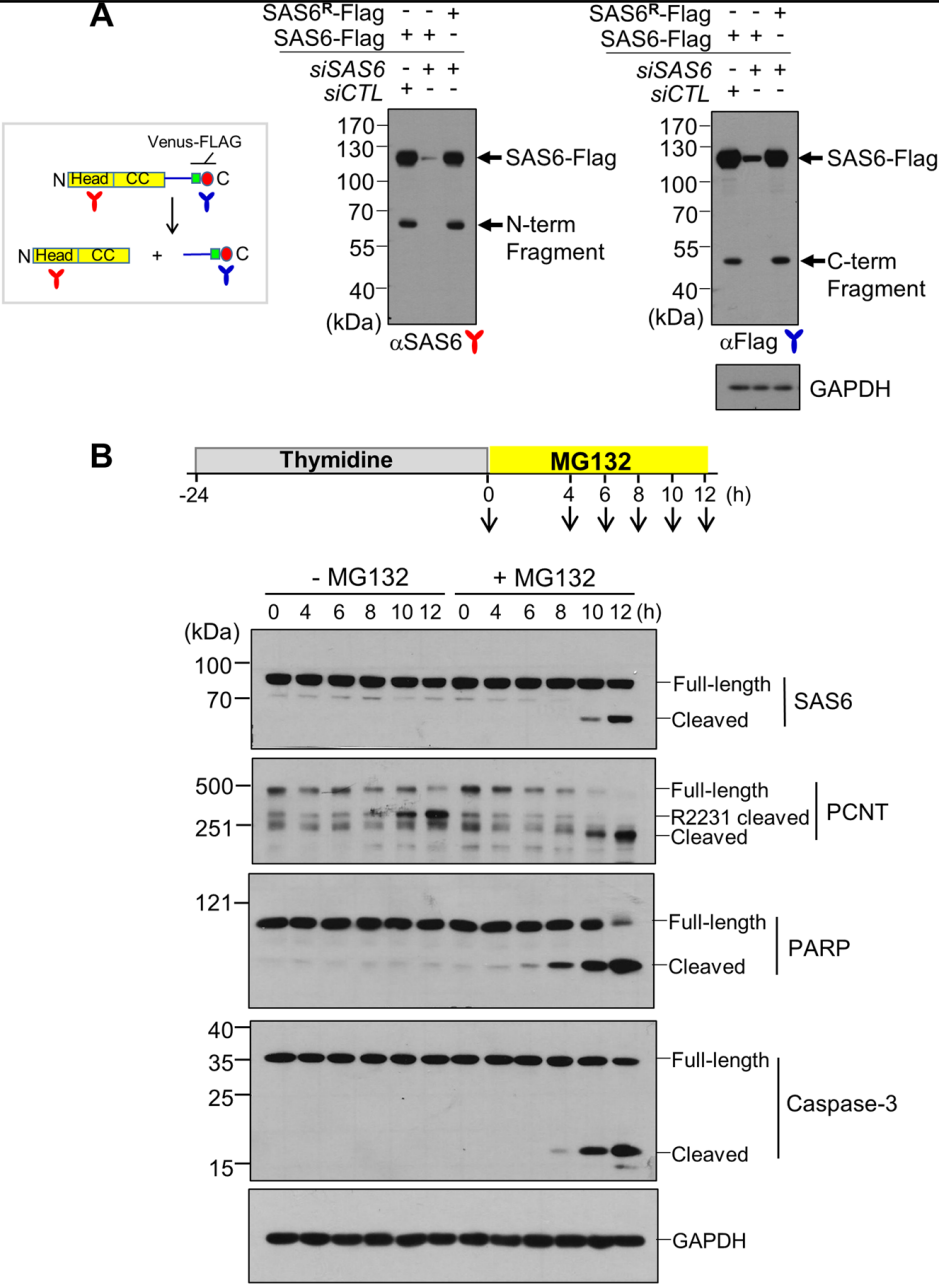
Detection of a specific cleavage of SAS-6. **a** HeLa cells were transfected with *pSAS-6-Venus-Flag*, along with *siCTL* or *siSAS-6*. SAS-6^R^-Flag includes silent mutations resistant to *siSAS-6*. Forty-eight hours later, the cells were subjected to immunoblot analyses with antibodies specific to SAS-6 and Flag. **b** HeLa cells were treated with thymidine for 24 h and transferred into a fresh medium with or without MG132. The cells were harvested at the indicated time points and subjected to immunoblot analyses with antibodies specific to SAS-6, pericentrin (PCNT), PARP-1, caspase-3, and GAPDH. Pericentrin cleavage normally occurs during mitotic exit^14^ and the cleavage band was labeled as R2231 cleaved

SAS-6 is composed of a globular domain at the N-terminus, a coiled-coil domain in the middle, and a disordered region at the C-terminus^3–5^. When the recombinant SAS-6 with the Venus-Flag tag at the 3′ end was ectopically expressed in HeLa cells, it generated an expected band of 115 kDa and an additional band of 65 kDa in size, which were detected with the SAS-6 antibody (Fig. 1a). When we performed the immunoblot analysis with the Flag antibody, we detected a band of 50 kDa in size along the intact protein band (Fig. 1a). The smaller bands disappeared along with the intact band by *siSAS-6* transfection, but reappeared when the siRNA target sites had been silently mutated (Fig. 1a). The immunoblot results suggest that the ectopic SAS-6-Venus-Flag protein can be cleaved near the end of the coiled-coil domain (Fig. 1a).

In order to detect the specific cleavage of endogenous SAS-6 during the cell cycle, we treated HeLa cells with thymidine for 24 h and transferred them into a fresh medium with or without MG132, a proteasome inhibitor. The results showed that the treatment of MG132 induced apoptosis, as exemplified by the presence of specific cleavage bands of PARP-1 and caspase-3 (Fig. 1b). The cleaved fragment of SAS-6 started to appear at 8–10 h only when the cells were treated with MG132 (Fig. 1b). Immunoblot analysis with the percentrin antibody generated multiple bands, in addition to a full-length band, which had been previously reported (Fig. 1b; ref. ^8^). We also observed a novel cleavage band of pericentrin during treatment of MG132 (Fig. 1b). In summary, we observed specific cleavage of SAS-6 and pericentrin in cells treated with MG132.

### Caspase-dependent cleavage of SAS-6 and pericentrin during apoptosis

**Fig. 2:**
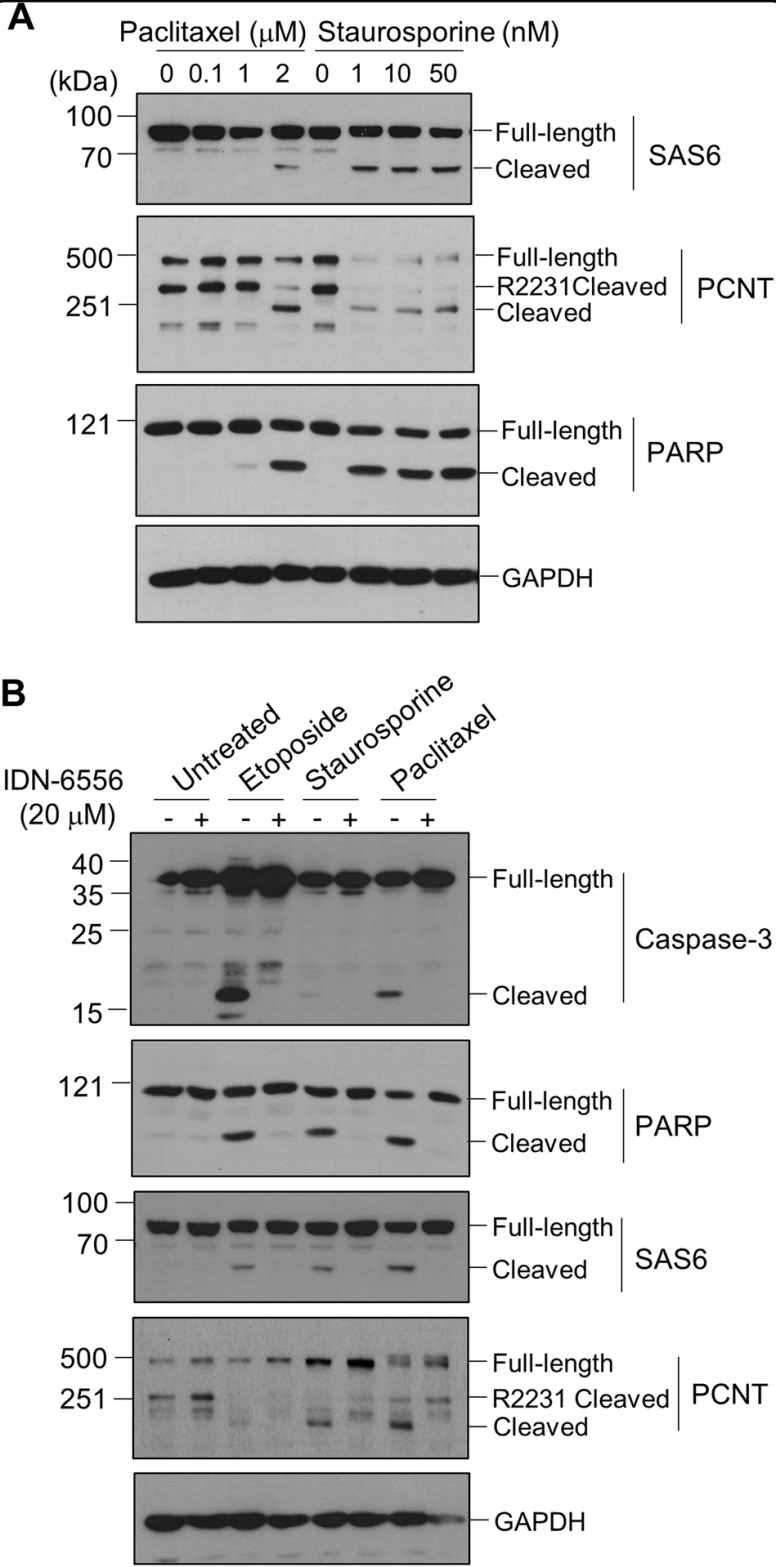
Caspase-dependent cleavage of SAS-6 and pericentrin during apoptosis. **a** HeLa cells were treated with paclitaxel or staurosporine for 24 h and subjected to immunoblot analyses with antibodies specific to SAS-6, pericentrin (PCNT), PARP-1, and GAPDH. **b** HeLa cells were treated with etoposide, staurosporine, or paclitaxel in the presence of IDN-6566 for 24 h. The cells were subjected to immunoblot analyses with indicated antibodies

Induction of apoptosis is one of the known effects of MG132^32–34^. It is also reported that the combination of cell-cycle arrest by thymidine and MG132 may accelerate the apoptotic processes^35^. Therefore, we investigated whether specific cleavages of SAS-6 and pericentrin were triggered by apoptosis-inducing agents, such as paclitaxel, staurosporine, and etoposide. The results showed that treatment of paclitaxel or staurosporine induced specific cleavage of PARP-1 in a dose-dependent manner (Fig. 2a). Under this condition, SAS-6 and pericentrin were also cleaved, suggesting that those proteins are specifically cleaved during apoptosis. Among the centrosomal proteins we examined, SAS-6 and pericentrin are the only two centrosomal proteins cleaved during apoptosis (Supplementary Fig. S1).

We next tested whether the inhibition of caspase activity might reduce the cleavage of the centrosomal proteins or not. HeLa cells were incubated with each apoptosis-inducing agent with or without IDN-6556, a pan-caspase inhibitor, for 24 h. As expected, the cleavages of PARP-1 and capase-3 were reduced with the caspase inhibitor (Fig. 2b). Specific cleavages of SAS-6 and pericentrin were also effectively diminished with the IDN-6556 treatment (Fig. 2b). This result suggests that the cleavages of SAS-6 and pericentrin are mediated by caspases.

### Determination of the specific cleavage site of SAS-6

**Fig. 3:**
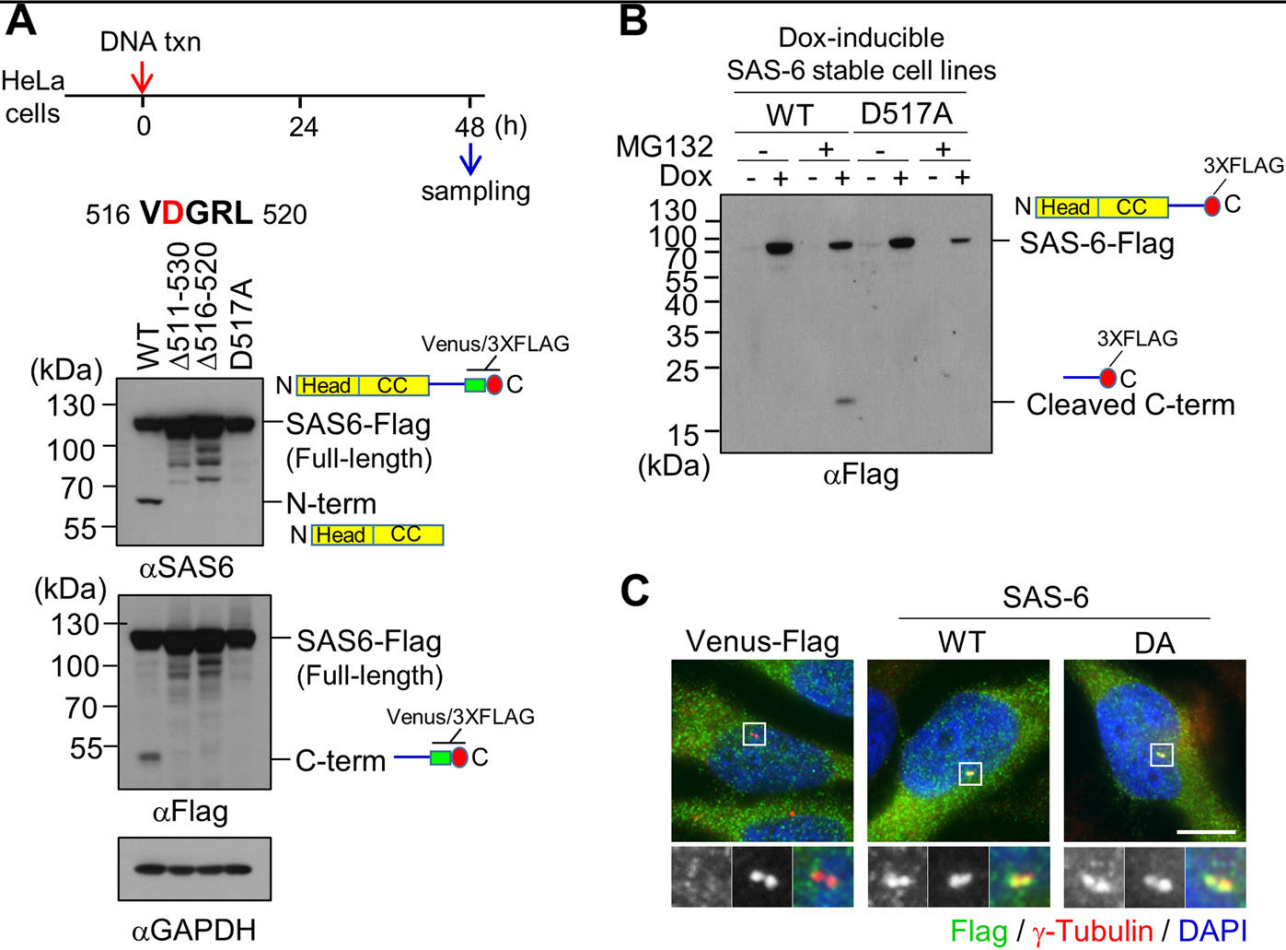
Identification of the specific cleavage site of SAS-6. **a** Immunoblot analysis was performed with HeLa cells expressing the wild type, the truncated (Δ511–530 and Δ516–520), or the substituted (D517A) mutant of SAS-6-Flag. The ectopic SAS-6 proteins were detected with antibodies specific to SAS-6 and Flag. **b** SAS-6-stable cell lines of the wild type and the D517A mutant. It is of note that the recombinant SAS-6 expresses a 3xFLAG at its C-terminus with the Venus tag removed. While the wild type generated a cleaved C-terminus fragment, the D517A mutant was resistant to the cleavage in the presence of MG132. **c** Immunostaining analysis of the wild type and the D517A mutant of ectopic SAS-6 proteins was performed with the Flag antibody. Scale bar, 10 μm

The previous immunoblot results suggest that SAS-6 cleavage occurs near the end of the coiled-coil domain (Fig. 1a). Using a series of the truncated mutants of SAS-6, we narrowed down the cleavage site to 516–520 residues (Fig. 3a; Supplementary Fig. S2). Alanine substitution of D517 made the SAS-6 mutant resistant to cleavages, suggesting that D517 residue is critical to the SAS-6 cleavage (Fig. 3a). In fact, the truncated mutants containing D517 residue of SAS-6 (Δ511–530 and Δ516–520) and the D517A mutant did not generate the cleaved fragments with the MG132 treatment (Fig. 3b, Supplementary Fig. S2C). However, the aspartate residue of human SAS-6 was not highly conserved among different species (Supplementary Fig. S3). Taken together, we concluded that the aspartate residue at 517 is the cleavage site of SAS-6 during apoptosis. We also confirmed the centrosomal localization of the D517A mutant of SAS-6 (Fig. 3c).

We also searched for potential cleavage sites of pericentrin via the ELM database^36^ and identified six candidate sites (Supplementary Fig. S4A). When we treated HeLa cells with STLC to arrest the cell cycle at M phase for prolonged time periods, we observed both separase-specific and caspase-specific cleavage bands of pericentrin (Supplementary Fig. S4B). The separase-specific cleavage band was absent when the pericentrin-depleted HeLa cells were rescued with the ectopic pericentrin with R2231A point mutation (Supplementary Fig. S4C). However, we still detected the caspase-specific cleavage band, suggesting that the separase-specific cleavage is not the prerequisite for the caspase-specific cleavage of pericentrin (Supplementary Fig. S4C). The caspase-specific cleavage band was not detectable with the Flag antibody, implying an additional cleavage site near the N-terminus of pericentrin (data not shown). It remains to be determined for the caspase-dependent cleavage sites of pericentrin and the proteases responsible for the multiple cleavages. We do not rule out the possibility that other kinds of proteases may be involved in the pericentrin cleavage.

### Effects of apoptotic agents on the centrosomal SAS-6 and pericentrin

**Fig. 4:**
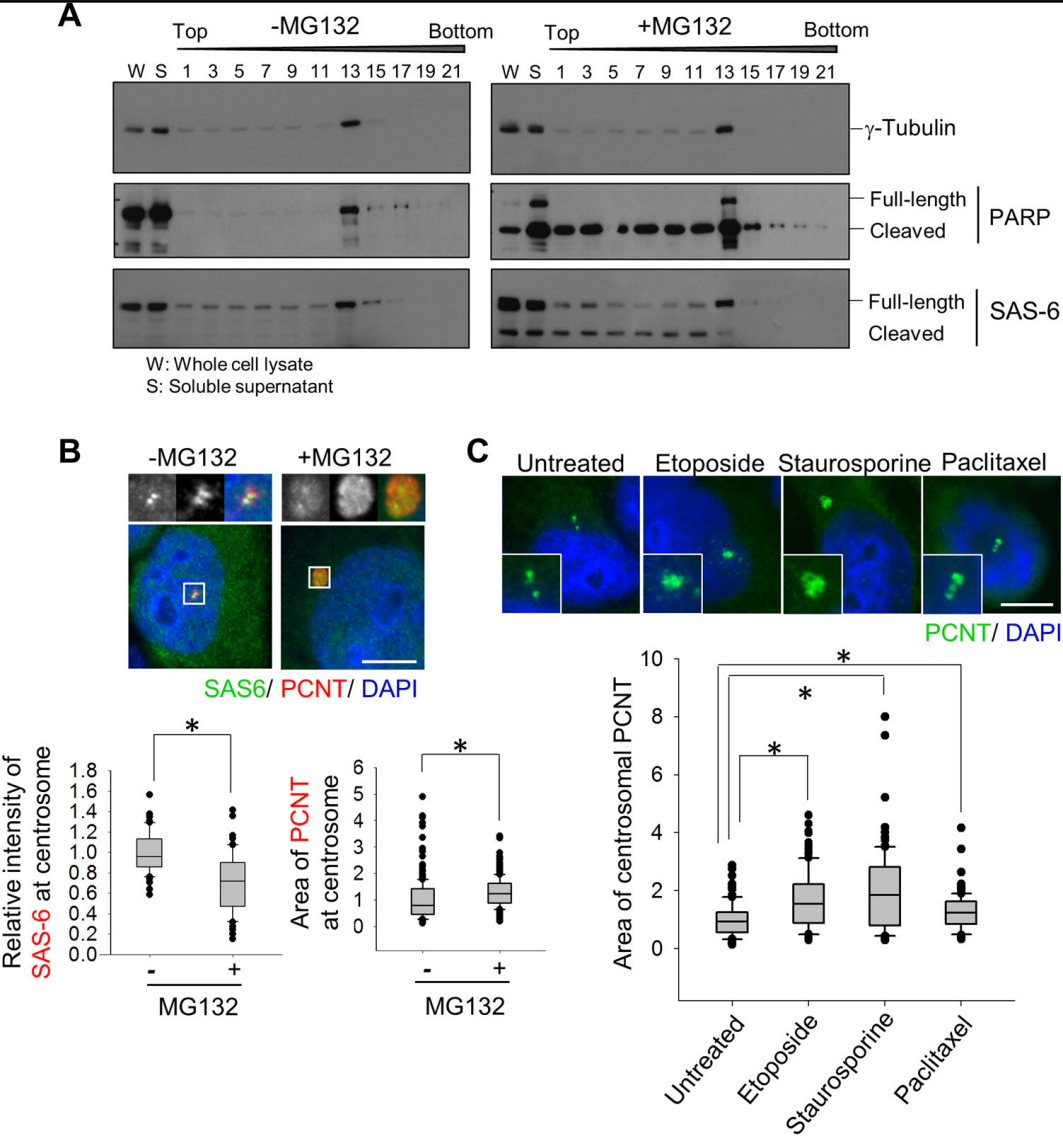
Effects of apoptotic agents on the centrosomal SAS-6 and pericentrin. **a** HeLa cells were treated with thymidine and transferred into a medium with MG132. Ten hours later, the cell lysates were fractionated with sucrose-gradient ultracentrifugation and subjected to immunoblot analyses with antibodies specific to γ-tubulin, PARP-1, and SAS-6. **b** The G2 phase HeLa cells were treated with MG132 for 8 h and coimmunostained with antibodies specific to SAS-6 (green) and pericentrin (red). DNA was visualized with DAPI (blue). Scale bar, 10 μm. The centriolar intensity of SAS-6 and centrosomal area of pericentrin were measured and presented with box and whisker plots. **c** HeLa cells were treated with etoposide, staurosporine, or paclitaxel for 24 h and immunostained with the pericentrin antibody. Greater than 100 centrosomes per experimental group were analyzed in two independent experiments. *P < 0.05

SAS-6 is the major component of procentrioles. However, a reserved pool of SAS-6 is also present in the cytoplasm. To test whether the centrosomal SAS-6 was cleaved or not, we enriched the centrosome fraction out of the cytosolic fractions, using the sucrose density-gradient ultracentrifuge. As shown in Fig. 4a, the centrosomes were enriched in fraction number 13 around 40–60% of sucrose density. The cleaved band of SAS-6 was detected in both the cytosolic and centrosomal fractions (Fig. 4a). We also observed centrosomal enrichment of PARP-1 in a normal state (Fig. 4a). Apoptosis-related PARP-1 cleavage was detected in both the centrosome and cytoplasm in the MG132-treated cells (Fig. 4a). The results suggest that SAS-6 cleavage occurs in both the centrosome and cytoplasm.

We examined the effects of MG132 on the centrosomal SAS-6 and pericentrin. The results showed that the MG132 treatment reduced the centrosomal levels of SAS-6. The centrosomal intensities of pericentrin were not significantly altered but the area of pericentrin was enlarged by the MG132 treatment (Fig. 4b). An increase in the pericentrin area was also detected with the treatment of apoptosis-inducing agents such as etoposide, staurosporine, and paclitaxel (Fig. 4c). Enlargement of the pericentrin area suggests that PCM is expanded in cells undergoing apoptosis.

### Reduction of the centrosomal SAS-6 levels in apoptotic cells

**Fig. 5:**
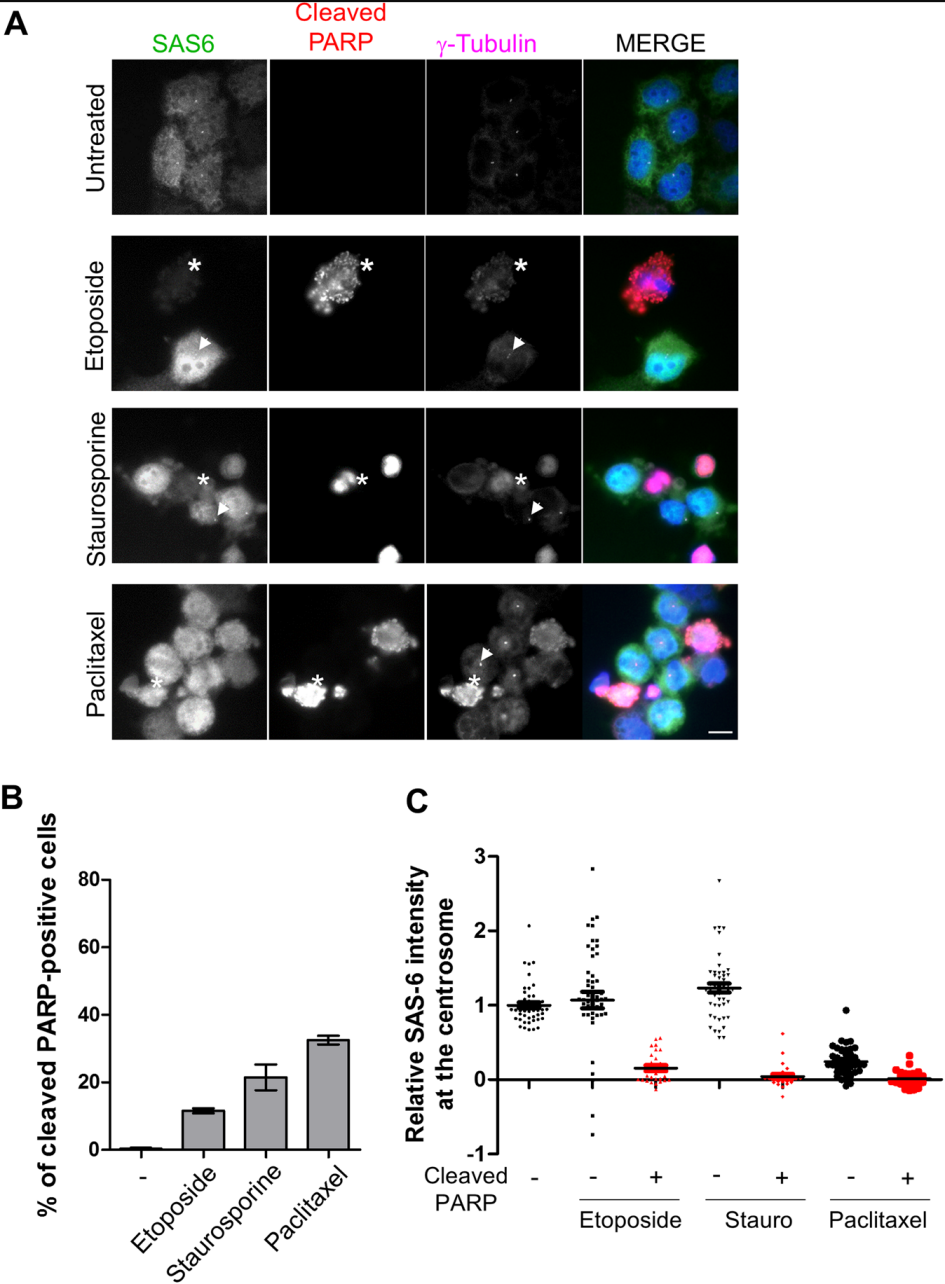
Reduction of the centriolar SAS-6 levels in cells undergoing apoptosis. **a** HeLa cells were treated with etoposide, staurosporine, or paclitaxel for 24 h and subjected to coimmunostaining with antibodies specific to SAS-6 (green), cleaved PARP-1 (red), and γ-tubulin (magenta). Asterisks indicate the cleaved PARP-positive cells whose SAS-6 intensities were significantly reduced. The arrowheads represent the cleaved PARP-negative cells with the remaining SAS-6 signals at the centrosomes. **b** Proportions of the cleaved PARP-1-positive cells were determined. **c** The centriolar intensities of SAS-6 were measured in cleaved PARP-1-negative or PARP-1-positive cells and analyzed with a scatter plot. Greater than 100 centrosomes per experimental group were analyzed in two independent experiments. *P < 0.05

We further analyzed the centrosomal SAS-6 in apoptotic cells, using the cleaved PARP-1 antibody. Population of the cleaved PARP-positive cells started to appear with the treatment of apoptosis-inducing agents, such as etoposide, staurosporine, and paclitaxel (Fig. 5a, b). The centrosomal levels of SAS-6 were almost completely suppressed in the cleaved PARP-positive cells (Fig. 5a, c). The same apoptosis-inducing agents incompletely suppressed the centrosomal levels of centrin-2 in the cleaved PARP-positive cells (Supplementary Fig. S5). These results suggest that complete removal of the centrosomal SAS-6 might be linked to the specific cleavage of SAS-6 during apoptosis.

### Rescue effects of the centrosomal SAS-6 levels by a caspase inhibitor

**Fig. 6:**
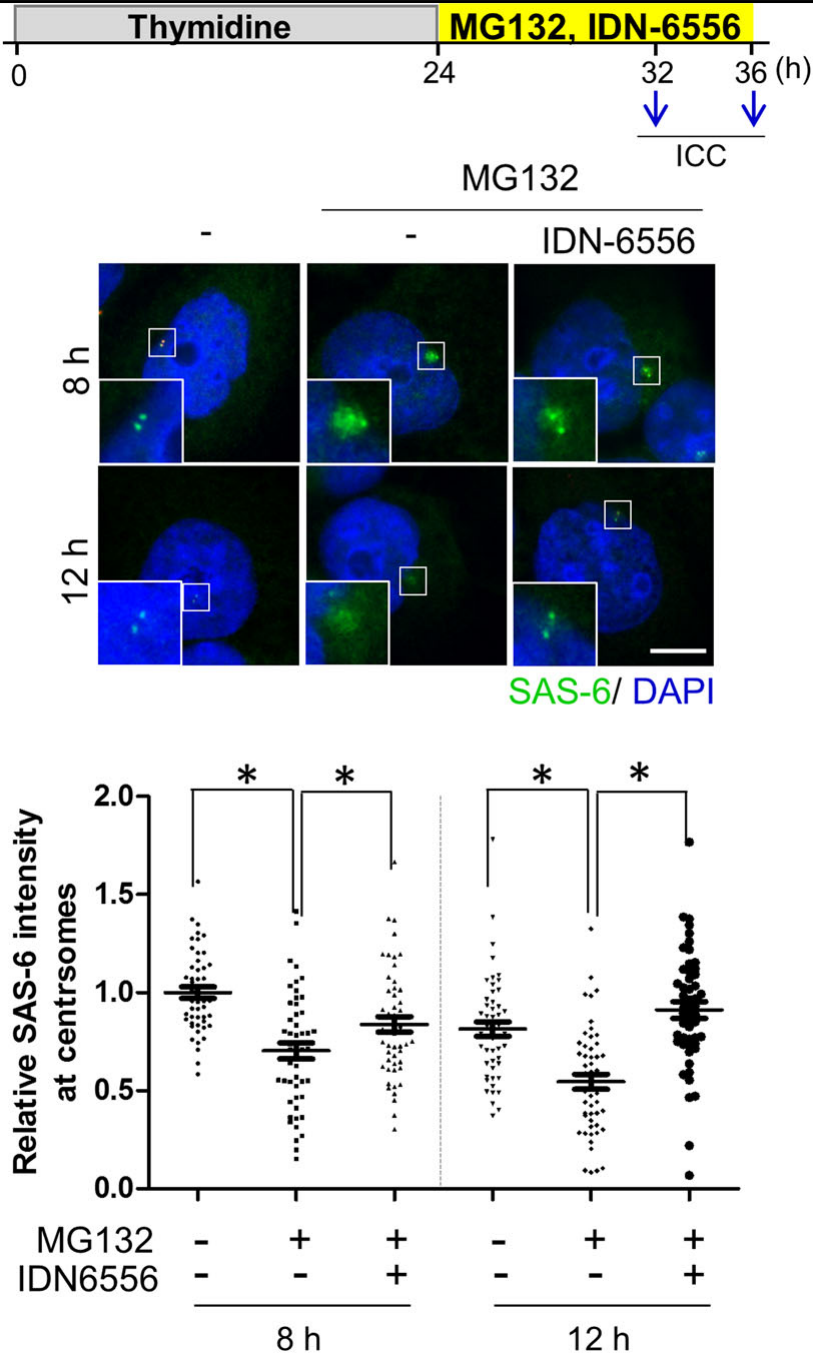
Effects of IDN-6556 on the centriolar SAS-6 levels in cells undergoing apoptosis. HeLa cells were treated with thymidine for 24 h, released into a fresh medium with MG132 and IDN-6566, and cultured for 8 or 12 h. The cells were immunostained with the SAS-6 (green) antibody. DNA was visualized with DAPI (blue). Scale bar, 10 μm. The centriolar intensity of SAS-6 was measured and analyzed with a scatter plot. Greater than 100 centrosomes per experimental group were analyzed in two independent experiments. *P < 0.05

In order to test whether the caspase activity is important for reduction of the centrosomal levels of SAS-6, we treated IDN-6556, a caspase inhibitor, along with MG132. The results showed that reduction of the centriolar SAS-6 intensity was repeated upon the inhibition of proteasome activity (Fig. 6). Treatment of IDN-6566 rescued the centriolar SAS-6 intensity. The effect of the caspase inhibitor was magnified when the cells were further incubated for four more hours (Fig. 6). The results support the notion that reduction of the centrosomal SAS-6 level might be an outcome of the SAS-6 cleavage mediated by caspases.

### Reduction of the centriole assembly activity under an apoptosis-inducing condition

**Fig. 7:**
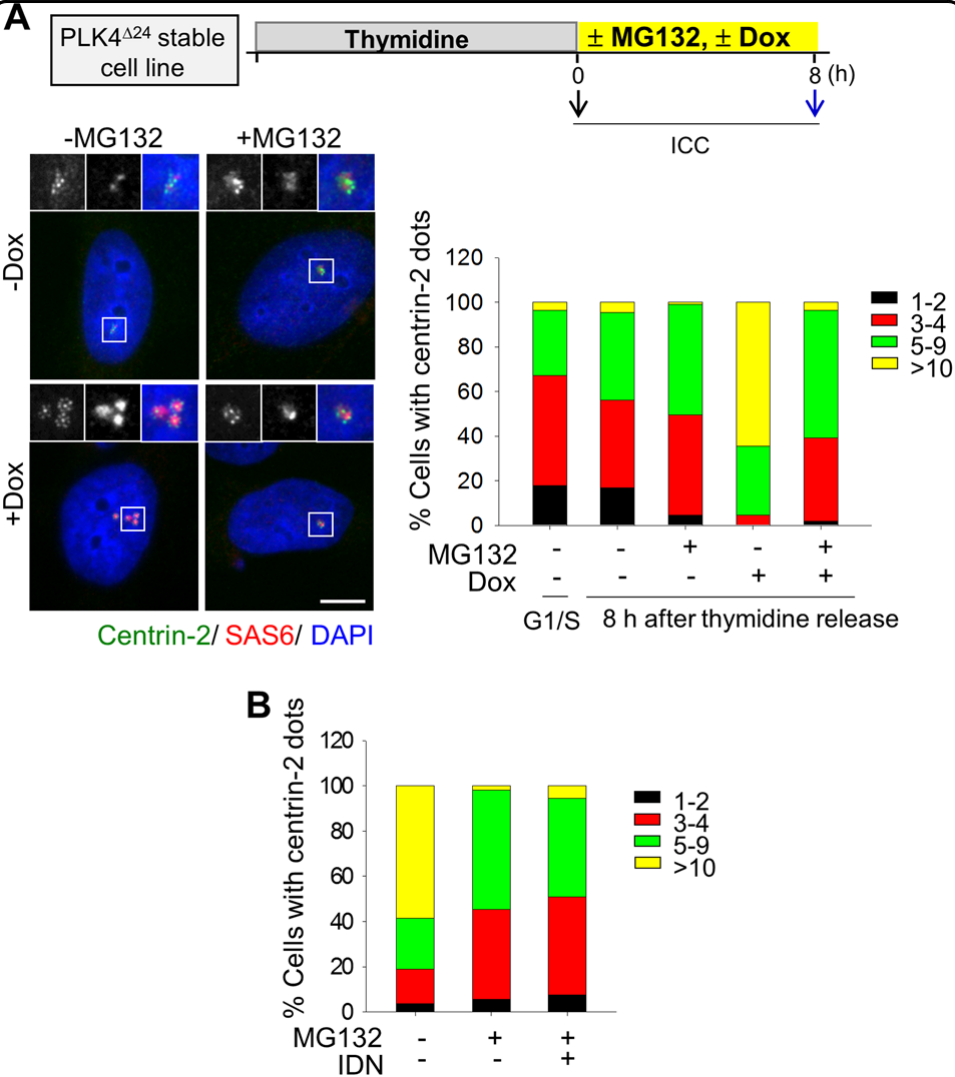
Inhibition of the proteasome activity affects the efficiency of centriole duplication in PLK4^Δ24^ cells. **a** HeLa cells were treated with thymidine for 24 h, and transferred into the medium with MG132. Expression of PLK4^Δ24^ was induced with doxycycline at the same time. At the indicated time points, the cells were coimmunostained with antibodies specific to centrin-2 (green) and SAS-6 (red). DNA was stained with DAPI (blue). Scale bar, 10 μm. The number of centrioles per cell were determined with the centrin-2 signals. **b** The PLK4^Δ24^-expressing cells were treated with IDN-6556 and their centriole numbers were counted. Greater than 100 cells per experimental group were analyzed in two independent experiments

SAS-6, the cartwheel protein, is essential for procentriole assembly^37^. Therefore, one can expect that reduction of the centrosomal SAS-6 levels might inhibit centriole assembly. We tested this possibility using a PLK4-overexpressing cell line. PLK4 is the major kinase for the initiation of centriole assembly^37,38^. Expression of the phosphodegron-removed mutant PLK4^Δ24^ could be induced by doxycycline^37,39–41^. The PLK4^Δ24^-expressing cells were synchronized at G1/S and released into a fresh medium with or without MG132 for 8 h. At the time of thymidine release, doxycycline was treated to induce centriole assembly by PLK^Δ24^. The cells were fixed in the indicated time points and coimmunostained with antibodies specific to centrin-2 and SAS-6. The number of centrioles was measured by quantifying the dot number of centrin-2. The results showed that the centriole assembly activity in PLK4^Δ24^-expressing cells was severely impaired with the MG132 treatment (Fig. 7a). However, such reduction was not relieved by IDN-6556, suggesting that the block of SAS-6 and pericentrin cleavages is not sufficient to rescue the efficiency of centriole assembly upon the MG132 treatment (Fig. 7b).

## Discussion

The present study reports specific cleavages of SAS-6 and pericentrin in apoptotic cells. Caspases are responsible for the cleavage. Specific cleavage of SAS-6 occurs at the centrosome as well as in the cytosol. This work demonstrates the first centrosomal targets of caspases during apoptosis.

Protein degradation is an important regulatory mechanism in the centrosome. A number of E3 ubiquitin ligases have been identified to control the centrosome cycle and ciliogenesis^42,43^. For example, SCF-Slimb/βTrCP E3 ubiquitin ligase tightly regulates the level of PLK4 by recognizing the conserved phosphodegron and therefore it is important for regulating centriole numbers^40,44^. SAS-6 is also known to be controlled by SCF–FBXW5 ubiquitin ligase to initiate centriole assembly during G1/S phase^45^. Neurl4-dependent regulation of CP110 level was shown to control cilia assembly^46^. Another mechanism for protein degradation may be separase-dependent cleavage of pericentrin, which is critical for centriole separation during mitotic exit^13,14^. In this study, we observed that a fraction of SAS-6 was cleaved when excessively expressed (Fig. 1). Specific cleavage of SAS-6 and pericentrin was obvious when the cells underwent apoptosis (Fig. 2). Therefore, we believe that the caspase-mediated cleavage is another example of protein-degradation mechanisms in the centrosome. It remains to be determined how caspases execute degradation of centrosome proteins under stressful conditions leading to cell death. Our preliminary results suggest that only a selective number of centrosomal proteins is cleaved by caspase-mediated pathways (Supplementary Fig. S1). Extensive proteomic approaches identified a few centrosomal proteins as potential substrates of caspases without validation^22,47^. Nonetheless, SAS-6 and pericentrin were not included in any database. As far as we know, this is the first evidence showing the link between caspases and centrosomal targets during apoptosis and the possible consequences of the cleavages of SAS-6 and pericentrin.

The most visible changes of the centrosomes in apoptotic cells may be the PCM expansion^48,49^. Such morphological change in apoptotic cells should be linked to reduction of microtubule-organizing activity of the centrosome^48,49^. We propose that caspase-dependent cleavage of pericentrin might confer PCM disorganization and expansion during apoptosis (Fig. 7). Eventually, pericentrin will be lost from the centrosome in apoptotic cells, when the cleavage event is dominant during the late phase of apoptosis^31^. With disorganized PCM, the microtubule-organizing activity should be impaired^48,49^. In fact, separase-dependent cleavage of pericentrin is known to confer disintegration of PCM during mitotic exit^15^. Of note, PCM expansion after DNA damage was also reported^50^. Checkpoint kinase 1 (CHK1), which is known to be a key player of DNA damage response pathway, also regulates pericentrin-dependent PCM expansion upon DNA damage by IR^50^. The marked expansion was supposed to be due to pericentrin cleavage. Collectively, these results suggest that PCM expansion might be a shared phenomenon in both DNA damage responses and chemical-induced apoptosis.

We also observed that the centrosomal SAS-6 levels were significantly reduced when the cells underwent apoptosis. Reduction of the centrosomal SAS-6 levels might be due to the direct cleavage of the cartwheel by caspases. Cleavage of the cytoplasmic SAS-6 by caspases might also contribute to the reduced SAS-6 intensity at the centrosome. Inferred from the PARP cleavage in the centrosome fraction (Fig. 4a), we suppose an active action of caspases at the centrosome. It remains to be investigated what are the direct outcomes of the reduced SAS-6 levels in the centrosome. One possibility is that reduction of the centrosomal SAS-6 level might be linked to the centriole assembly activity^3,51^. In fact, we observed that the centriole assembly activity was reduced when the cells were treated with MG132 for sufficient time to induce SAS-6 cleavage (Fig. 7). Caspase-specific cleavage of SAS-6 might also cause structural defects of daughter centriole with a reduced cartwheel structure. Indeed, the centriolar markers represent the mother centrioles remained intact, while the daughter centrioles with SAS-6 signals disappeared in cells treated with paclitaxel.

**Fig. 8:**
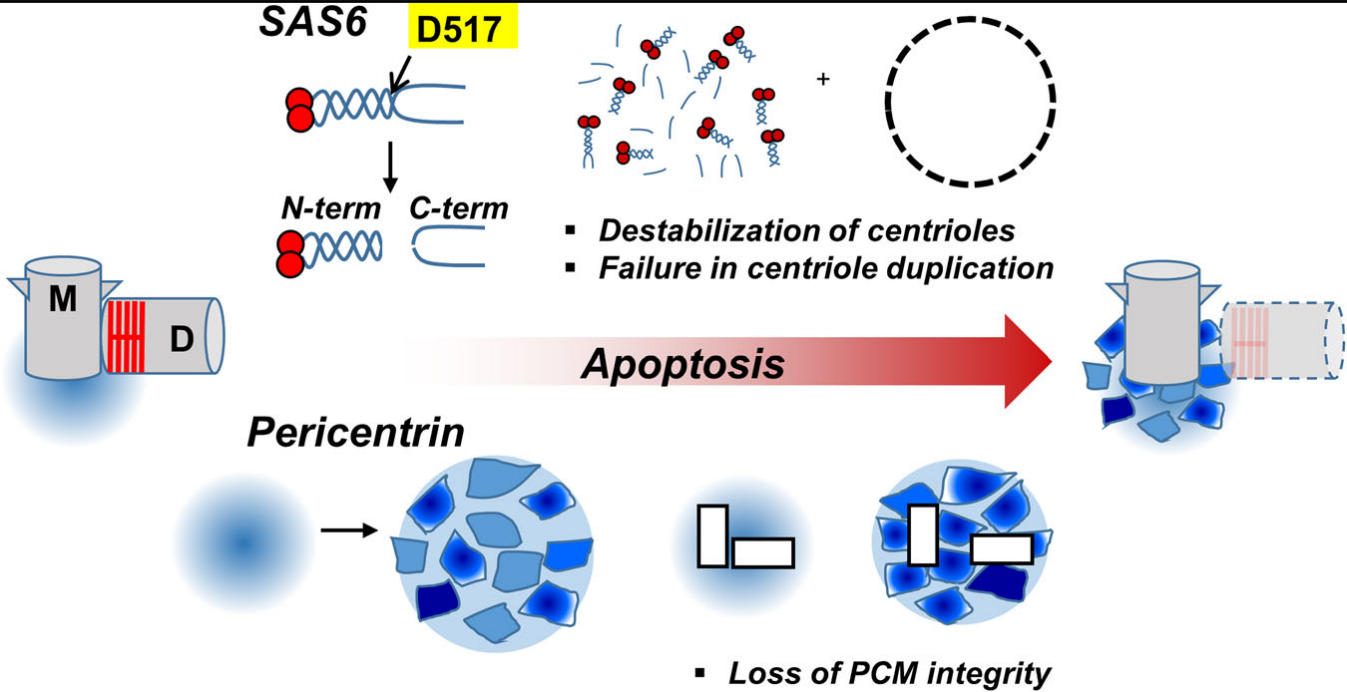
Model. During apoptosis, selected centrosomal proteins, such as SAS-6 and pericentrin, are cleaved by caspases. The specific cleavage of SAS-6 might destabilize the procentrioles and prevent a new centriole assembly. Caspase-dependent cleavage of pericentrin might attribute to loss of PCM integrity

The centrosome serves as a scaffold for efficient microtubule assembly under normal conditions. During apoptosis, its microtubule-organizing activity is limited, probably due to caspase-mediated cleavage of the centrosomal proteins. Instead, apoptotic microtubule networks (AMN) assemble independently of the centrosome^31,52,53^. The centrosome might inhibit the formation of an extensive bundle of AMN, which facilitate the apoptotic processes during the execution phase. Thus, specific caspase-mediated cleavage of the centrosomal proteins may be a prerequisite for the formation of AMN (Fig. 8).

It is known that caspases are also involved in non-apoptotic processes. For example, caspase-2, 3, or 9 is required for myoblast differentiation^54–56^. Dissolution of centrosomes is accompanied with this process^57^. It may be interesting to determine whether SAS-6 and pericentrin are cleaved by caspases during myoblast differentiation.

This paper reports that caspase-specific cleavage of SAS-6 and pericentrin might be linked to the functional disruption of the centrosome during apoptosis. We currently investigate direct outcomes of cleavage of SAS-6 and pericentrin on the centrosome morphology and functions during apoptosis.

## Materials and methods

### Cell culture, drug treatment, and plasmids

HeLa cells were cultured in DMEM supplemented with 10% fetal bovine serum at 37 ˚C and 5% CO_2_. Cells were synchronized with a single thymidine block and released into the medium with or without MG132 (20 μM). To induce apoptosis, apoptosis-inducing drugs such as etoposide (50 μM), straurosporine (100 nM), or taxol (2 μM) were treated for 24 h. IDN-6556, an inhibitor of caspases was treated at 20 μM.

The human SAS-6 cDNA clone was subcloned into the pcDNA5 FRT/TO (hygromycin) vector. The siRNA-resistant constructs of SAS-6 were generated by site-directed mutagenesis. The sequence of siRNA-resistant SAS-6 is as follows: target SAS-6 sequence, G CAC(His) GTT(Val) AAT(Asn) CAG(Gln) CTA(Leu) CAA(Gln) TT; siRNA-resistant construct, G CAT(His) GTG(Val) AAC(Asn) CAA(Gln) CTC(Leu) CAA(Gln) TT. Plasmids were transfected with FuGENE HD (Roche). For inducible expression of the ectopic SAS-6 wild type and the truncated mutants, HeLa Flp-In TREX cell line (a kind gift from Dr. Stephen Taylor) was stably transfected with the constructs and selected with hygromycin (0.4 mg/ml).

The human *PLK4* cDNA clone was also subcloned into the pcDNA5 FRT/TO (hygromycin) vector and later the phosphodegron was deleted from the full-length PLK4 to avoid the proteosomal degradation by MG132. To establish a PLK4^Δ24^-expressing cell line, HeLa Flp-In TREX cell line was also used for stable transfections. To induce centriole duplications, PLK4^Δ24^-stable cell line was treated with doxycycline (1 μg/ml).

### siRNAs

The siRNAs were purchased (ST Pharm, Korea) and the sequences are as follows: *siCTL* (scrambled sequence for control) (GCA AUC GAA GCU CGG CUA C-dTdT), *siSAS6* (GCA CGU UAA UCA GCU ACA A-dTdT), and *siPCNT* (UGG ACG UCA UCC AAU GAG A-dTdT). The siRNAs were transfected into cells using RNAi MAX reagents (Invitrogen).

### Antibodies

Anti-rabbit polyclonal antibodies against pericentrin, CPAP, and CEP215 were previously generated in-house as reported^58,59^. The antibodies against γ-tubulin (sc-7396, Santa Cruz Biotechnology, Inc.), centrin-2 (04-1624, Millipore), Flag (F3165, Sigma), SAS-6 (sc-376836, Santa Cruz Biotechnology, Inc.), STIL (ab89314, Abcam), CEP295 (ab122490, Abcam), and CEP152 (ab183911, Abcam) were purchased. For detection of apoptosis, anti-caspase-3 antibodies (9662, Cell Signaling), anti-PARP-1 antibodies (9542, Cell Signaling), and anti-cleaved PARP-1 antibodies (5625, Cell Signaling) were also purchased. Additional antibodies used for immunoblotting include the antibodies against GAPDH (AM4300, Ambion). HRP-conjugated secondary antibodies (A9044, Sigma and AP132P, Millipore) were used at 1:10,000 dilutions.

### Immunoblotting

For immunoblot analysis, the cells were first washed with PBS and lysed with lysis buffer (50 mM Tris, pH 7.5, 150 mM NaCl, 0.5% NP-40, 0.5% Triton X-100, 20 mM NaF, 20 mM β-glycerophosphate, and 1 mM Na_3_VO_4_) containing protease inhibitor cocktail (Sigma) for 30 min on ice. After centrifugation, the soluble fraction was used for protein quantification by Bradford assay. For each tested group, 15–25 μg of proteins were boiled with the Laemmli sample buffer and subjected to SDS-PAGE. After transferring onto a nitrocellulose membrane, it was blocked in 5% skimmed milk in TBST (20 mM Tris-Cl, pH 8.0, 150 mM NaCl, and 0.1% Tween 20) for 1 h, and incubated with the primary antibodies overnight at 4 °C. The membrane was then washed thrice with TBST. After incubation with HRP-conjugated secondary antibodies for 1 h, the membrane was washed thrice with TBST and visualized with ECL solutions.

### Immunofluorescence microscopy

For indirect immunocytochemistry, HeLa cells were grown on 12-mm coverslips and fixed with cold methanol for 10 min. The fixed cells were then blocked with PBS containing 3% BSA and 0.5% Triton X-100 for 15 min and incubated with the primary antibodies for 1–2 h at room temperature. After washing with 0.1% PBST, the cells were further incubated with the secondary antibodies conjugated with Alexa Fluor-488, 594, or 647 (Invitrogen; 1:10,000 dilutions). DNA was counterstained with DAPI solution. The samples were subsequently mounted in ProLong Gold antifade reagent (Invitrogen) and observed with a fluorescence microscope (Olympus IX51) equipped with a CCD camera (Qicam Fast 1394, Qimaging). The acquired images were processed using ImagePro 5.0 (Media Cybernetics, Inc.) for contrast adjustment and Photoshop 6.0 (Adobe) for image sizing.

### Centrosome enrichment

To enrich the centrosomes, HeLa cells were grown on 10-cm dishes. At 40–60% confluency, the cells were synchronized with 2 mM thymidine for 24 h and released into the medium with or without MG132 (20 μM) for 9 h. To dissociate the centrosome from the nucleus, the cells were incubated with 5 μg/ml cytochalasin D and 5 μg/ml nocodazole for 1 h. To enrich the centrosomes, cell lysis was performed in hypotonic solution as described in ref. ^60^. Then, the lysate was homogenized using a 15-ml dounce homogenizer. After removing the nucleus fraction by centrifugation, the supernatant was collected in a tube containing a final concentration of Triton X-100 at 0.1%. After centrifugation at 3000 rpm at 4°C for 5 min, the supernatant was then added carefully to the discontinuous sucrose gradient layered by 2.5 ml of 70% sucrose, 1 ml of 50% sucrose, and 1 ml of 40% sucrose in a 14-ml thin ultracentrifuge tube. Then the tubes were centrifuged at 30,000 rpm in a rotor at 4 °C for 1 h. Fractions from the top of the tube were collected and run on a SDS-PAGE for immunoblotting analyses.

## Electronic supplementary material

Supplementary Figures S1–S5

Supplementary figure legends
