# Supplementary material for: Caspase-mediated cleavage of the centrosomal proteins during apoptosis
Source: Cell Death Dis. 2018 May 11;9(5):571. doi: 10.1038/s41419-018-0632-8 (PMC5948218; doi:10.1038/s41419-018-0632-8)
Supplement: Supplementary file 1 — Supplementary Figures S1–S5 [file 41419_2018_632_MOESM1_ESM.pdf]

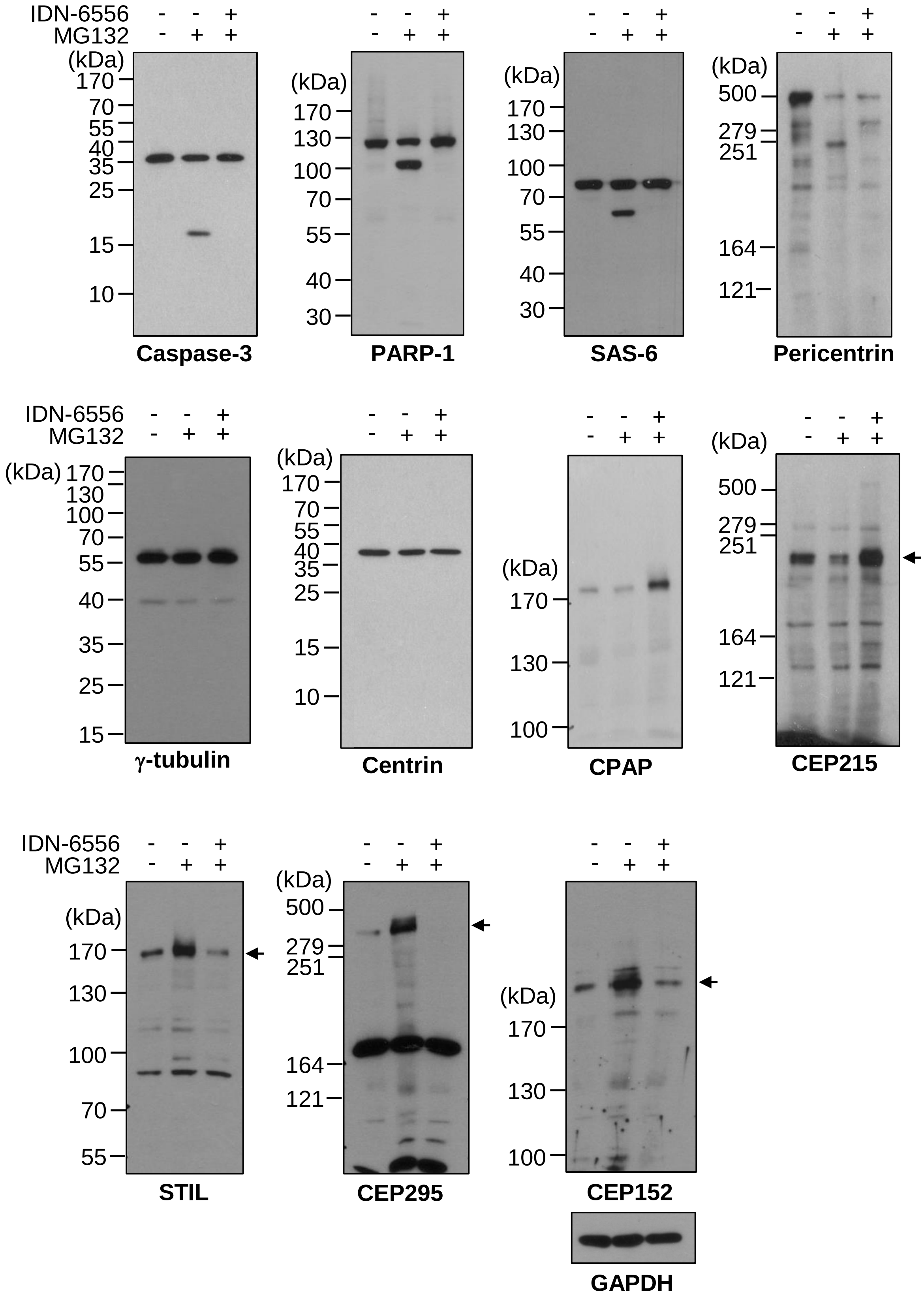

Supplementary Figure S1

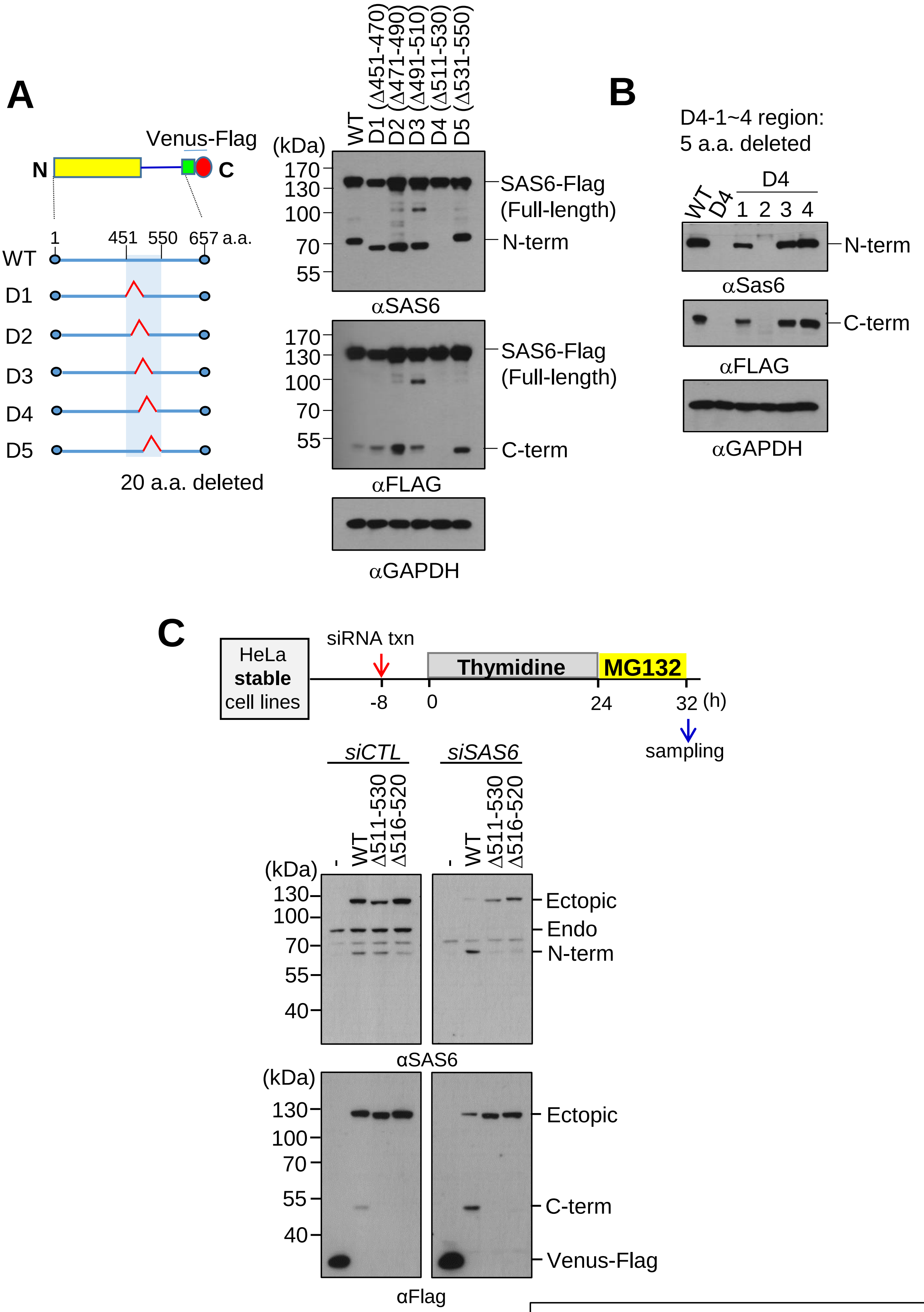

Supplementary Figure S2

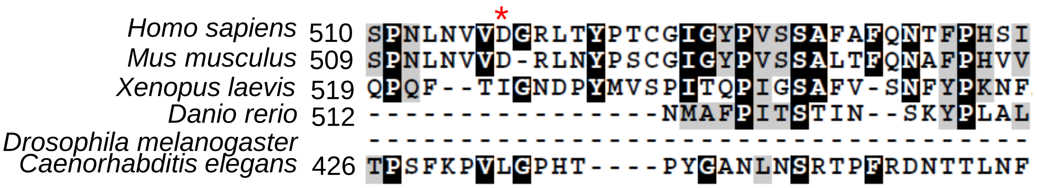

Supplementary Figure S3

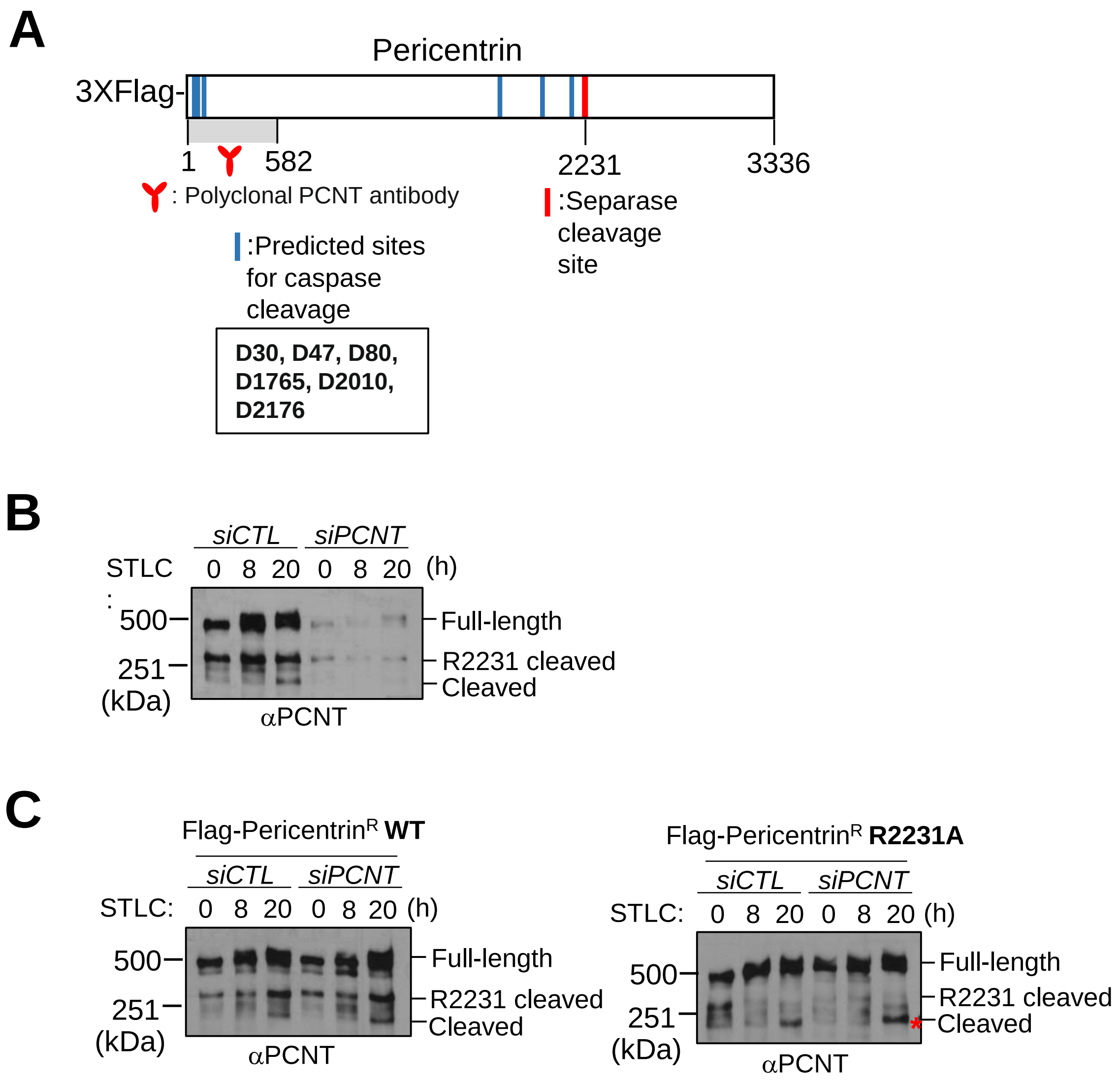

Supplementary Figure S4

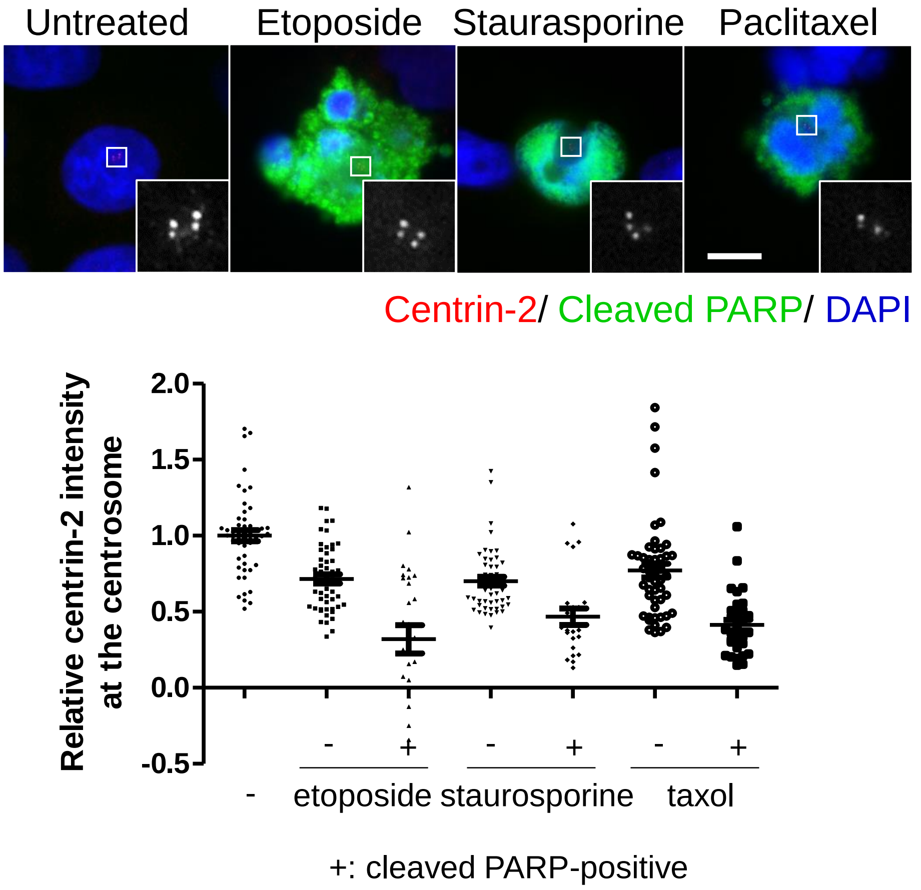

Supplementary Figure S5
