# Supplementary material for: Caspase-mediated cleavage of the centrosomal proteins during apoptosis
Source: Cell Death Dis. 2018 May 11;9(5):571. doi: 10.1038/s41419-018-0632-8 (PMC5948218; doi:10.1038/s41419-018-0632-8)
Supplement: Supplementary file 2 — Supplementary figure legends [file 41419_2018_632_MOESM2_ESM.docx]

**Supplementary Figure S1. Investigation into the cleavages of other centrosomal proteins.** SAS-6 and pericentrin are cleaved, while other centrosomal proteins remain intact. HeLa cells were synchronized with thymidine and released into the medium containing MG132. IDN-6556 treatment allows to discriminate a cleaved fragment by caspase from a non-specific one.

**Supplementary Figure S2. Strategy for identifying the specific cleavage site of SAS-6.** (A) A presumptive region for SAS-6 cleavage was first selected to span from 450 – 550 a.a. The region was then divided into 5 groups, each containing 20 a.a. deletion. The truncated mutants were subjected to immunoblot analysis with antibodies specific to SAS-6, Flag and GAPDH. (B) The D4 region was further divided into 4 groups, each containing deletions of 5 a. a. residues. Specific cleavage of the truncated mutants was analyzed with immunoblotting. (C) Stable cell lines of the wild type and the truncated mutants (D4, D4-2) of SAS-6 were examined for the generation of cleaved fragments. Endogenous SAS-6 was depleted with siRNA transfection in these cells, which stably expressed the siRNA-resistant SAS-6 proteins. The cells were treated with thymidine for 24 h followed by MG132 for 8 h, and subjected to immunoblot analysis with antibodies specific to SAS-6 and Flag.

**Supplementary Figure S3.** **Multiple sequence alignment of SAS-6.** The representative image shows the result of sequence alignment including D517 residue of human SAS-6

**Supplementary Figure S4. Analysis of potential cleavage sites of pericentrin. (**A) Predicted cleavage sites by caspases. The blue line shows the predicted sites for caspase cleavages (36). The red line indicates the known site (R2231) for separase cleavage. Our pericentrin antibody can recognize 1-582 a.a. of pericentrin. (B) When HeLa cells were cell cycle arrested for a long time, the cleaved fragment accumulated. Both the R2231 cleaved and the cleaved fragments disappeared with *siPCNT.* (C) Stable cell lines of the wild type and R2231A (non-cleavable) mutant of pericentrin were transfected with *siCTL* or *siPCNT*. The endogenous pericentin was assumed to be depleted as shown in panel B. The remaining pericentrin in *siPCNT*-transfected groups indicates the siRNA-resistant Flag-PCNT^R^ of the WT and the RA. R2231 cleaved fragment disappeared only in R2231A mutant cell line. However, the cleaved fragment still appeared in the RA mutant cell line. The red asterisk indicates the apoptotic fragment of PCNT.

**Supplementary Figure S5. Centriolar intensities of centrin-2 in cleaved PARP-positive cells.** (A) HeLa cells were treated with etoposide, staurosporine, or paclitaxel for 24 h and subjected to immunostaining with antibodies specific to centrin-2 (red) and cleaved PARP-1 (green). The centriolar intensities of centrin-2 were measured in cleaved PARP-1-negative and positive cells and analyzed with a scatter plot. Greater than 50 centrosomes were analyzed in two independent experiments. *, P < 0.05.
